# Genome-Wide Association Study Identifies Candidate Genes Related to Seed Oil Composition and Protein Content in *Gossypium hirsutum* L.

**DOI:** 10.3389/fpls.2018.01359

**Published:** 2018-10-22

**Authors:** Yanchao Yuan, Xianlin Wang, Liyuan Wang, Huixian Xing, Qingkang Wang, Muhammad Saeed, Jincai Tao, Wei Feng, Guihua Zhang, Xian-Liang Song, Xue-Zhen Sun

**Affiliations:** ^1^State Key Laboratory of Crop Biology/Agronomy College, Shandong Agricultural University, Taian, China; ^2^Department of Botany, Government College University, Faisalabad, Pakistan; ^3^Heze Academy of Agricultural Sciences, Heze, China

**Keywords:** cottonseed oil, fatty acid, seed protein, genome-wide association study, SNP, quantitative trait loci, cottonseed composition

## Abstract

Cotton (*Gossypium* spp.) is a leading natural fiber crop and an important source of vegetable protein and oil for humans and livestock. To investigate the genetic architecture of seed nutrients in upland cotton, a genome-wide association study (GWAS) was conducted in a panel of 196 germplasm resources under three environments using a CottonSNP80K chip of 77,774 loci. Relatively high genetic diversity (average gene diversity being 0.331) and phenotypic variation (coefficient of variation, CV, exceeding 3.9%) were detected in this panel. Correlation analysis revealed that the well-documented negative association between seed protein (PR) and oil may be to some extent attributable to the negative correlation between oleic acid (OA) and PR. Linkage disequilibrium (LD) was unevenly distributed among chromosomes and subgenomes. It ranged from 0.10–0.20 Mb (Chr19) to 5.65–5.75 Mb (Chr25) among the chromosomes and the range of Dt-subgenomes LD decay distances was smaller than At-subgenomes. This panel was divided into two subpopulations based on the information of 41,815 polymorphic single-nucleotide polymorphism (SNP) markers. The mixed linear model considering both Q-matrix and K-matrix [MLM(Q+K)] was employed to estimate the association between the SNP markers and the seed nutrients, considering the false positives caused by population structure and the kinship. A total of 47 SNP markers and 28 candidate quantitative trait loci (QTLs) regions were found to be significantly associated with seven cottonseed nutrients, including protein, total fatty acid, and five main fatty acid compositions. In addition, the candidate genes in these regions were analyzed, which included three genes, *Gh_D12G1161, Gh_D12G11*62, and *Gh_D12G1165* that were most likely involved in the control of cottonseed protein concentration. These results improved our understanding of the genetic control of cottonseed nutrients and provided potential molecular tools to develop cultivars with high protein and improved fatty acid compositions in cotton breeding programs through marker-assisted selection.

## Introduction

Cotton (*Gossypium* spp.) is the leading natural fiber crop for the manufacture of textiles and an important source of vegetable oil and protein for humans and livestock (Yu et al., 2012; Gore et al., 2014; Liu et al., 2017a), as well as renewable raw materials for various industrial products such as biofuels, lubricants, and hydraulic oils (Jiao et al., 2013; Sinha and Murugavelh, 2016). Cottonseed oil and protein account for 17–27% and 12–32% of seed weight, respectively, and vary with cotton species, varieties, and measuring methods (Wu et al., 2009; Yu et al., 2012). The fatty acids of cottonseed oil generally contain 22% saturated fatty acids and 74% unsaturated fatty acids (15% monounsaturated fatty acids and 59% polyunsaturated fatty acids) (Stewart et al., 2010). Cottonseed protein content, oil content, and its composition determine its nutritional values and physicochemical properties (Stewart et al., 2010; Lu et al., 2011). Therefore, improving the seed nutrients and its composition is an important cotton breeding target to increase the total output of cotton production.

Oil and protein content in cottonseeds are complex quantitative traits that are controlled by a series of genes with small effects and influenced by the environment (Hanny et al., 1978; Wu et al., 2009; Yu et al., 2012; Liu et al., 2015b). For cottonseed oil and protein content, in some studies, general and special combining abilities, maternal effects, and both additive and non-additive (including dominance) effects have been reported (Kohel, 1980; Dani and Kohel, 1989; Wu et al., 2009, 2010), whereas only additive effects were detected in other studies (Wu et al., 2009, 2010), indicating the complicated genetic architecture underlying these traits. Previous reports have shown a significant negative correlation between oil and protein concentration in cottonseed, thereby limiting the potential to develop cultivars with high seed oil and protein simultaneously in conventional breeding programs (Yu et al., 2012; Liu et al., 2015b).

Molecular markers have been used to explore QTLs (Quantitative Trait Loci) or chromosome regions conferring seed nutrients and composition in oilseed crops, including *Glycine max* (L.) Merrill (Reinprecht et al., 2006), *Brassica napus* L. (Javed et al., 2016), *Arachis hypogaea* L. (Shasidhar et al., 2017), and *Gossypium hirsutum* L. (Song and Zhang, 2007; Yu et al., 2012; Liu et al., 2015a), through linkage mapping approaches. A total of 64 significant QTLs for six seed nutrient traits (the crude oil, crude protein, linolenic acid, stearic acid, oleic acid, and palmitic acid content) were identified using a high-density upland cotton genetic map in a *G. hirsutum* intraspecific RIL population (Liu et al., 2015a). Additional cottonseed-related QTLs were identified in interspecific populations partly due to relatively high map coverage and marker resolution (Song and Zhang, 2007; Yu et al., 2012). In a *G. hirsutum* × *G. barbadense* BC_1_S_1_ population, three major QTLs controlling kernel percentage, kernel oil percentage, and kernel protein percentage were identified (Song and Zhang, 2007). Through an interspecific hybrid backcross inbred line population between *G. hirsutum* and *G. barbadense*, 42 QTLs (17 QTLs related to oil content, 22 QTLs for protein content, and three QTLs for gossypol content) were detected (Yu et al., 2012). In cottonseed, 56 QTLs for nine amino acid raw materials of protein synthesis were detected in two upland cotton populations (Liu et al., 2017b). However, among these cottonseed QTLs, only a few were identified in multi-environments or multiple genetic-background, and none have been widely used in marker-assisted selection (MAS) strategy of cotton breeding programs for high protein and/or oil. This might be caused by QTL population specificity, large QTL confidence intervals, QTL × genetic background, and QTL × environment interactions, which hinder the application of QTL in practical breeding (Mackay and Powell, 2007; Cavanagh et al., 2008; Qi et al., 2011). Thus, more loci for cottonseed nutrient traits need to be explored in diverse genetic backgrounds with different methods.

Association mapping, which is a complementary approach for setting up the genetic basis of quantitative traits, identifies QTLs on the basis of recombination events that occurred during the evolution of a panel of diverse germplasms and therefore provides the advantage of dissecting larger numbers of alleles than linkage mapping (Yu and Buckler, 2006; Rafalski, 2010; Tian et al., 2011; Saïdou et al., 2014). Genome-wide association studies (GWAS) have been widely adopted to analyze the genetic architecture of seed protein, oil, and fatty acid composition in oil crops, including soybean (Cao et al., 2017), rapeseed (Gacek et al., 2017), and sesame (Li et al., 2014a), and other plants such as maize (Tian et al., 2011; Li et al., 2013a) and *Arabidopsis thaliana* (Branham et al., 2015). For cotton, an association analysis using 228 simple sequence repeats (SSR) markers in a panel of 180 elite upland cotton cultivars and breeding lines detected 86 marker-trait (seed oil and protein content) associations in six environments (Liu et al., 2015b). Twenty-one QTLs for seed quality traits (protein, oil, and fiber content) were detected through GWAS in a panel of 75 upland genotypes with 234 polymorphic amplified fragment length polymorphisms (AFLPs) (Badigannavar and Myers, 2015). With the release of complete whole-genome sequences of the tetraploid cottons, *G. barbadense* (Liu et al., 2015c; Yuan et al., 2015) and *G. hirsutum*, (Li et al., 2015; Zhang et al., 2015) and the diploid cottons, *G. arboreum* (Li et al., 2014b) and *G. raimondii* (Paterson et al., 2012), GWAS using SNP data obtained by genotyping-by-sequencing (GBS) and genotyping array technologies has been undertaken to dissect the genetic regulation of complex traits in cotton, such as fiber quality, fiber yield, agronomy traits, salt, and verticillium wilt resistance (Islam et al., 2016; Cai et al., 2017; Fang et al., 2017; Huang et al., 2017a; Li et al., 2017; Sun et al., 2017; Wang et al., 2017). However, to our knowledge, no GWAS on cottonseed protein, oil, and fatty acid composition has been reported to date.

In this study, GWAS for seed protein, oil, and fatty acid composition was performed in a panel of 196 upland cotton accessions under three environments using genotypic data of 41,815 SNP markers from the Illumina CottonSNP80K (Cai et al., 2017). The objectives of this study were (i) to evaluate variations in seed protein, oil, and fatty acid composition in this panel of upland cotton accessions; (ii) to explore the genetic structure and linkage disequilibrium (LD) level in this panel; and (iii) to identify candidate QTL regions and genes conferring cottonseed oil and protein to facilitate the dissection of the genetic architecture of these important traits in upland cotton.

## Materials and methods

### Plant materials and field experiments

A panel of 196 diverse upland cotton accessions was selected for this GWAS, which originated from 11 countries in five continents (Table S1). This panel contained 169 accessions cultivated in China and 27 exotic accessions. The accessions cultivated in China were selected from the five cotton-growing regions in China: 139 genotypes from the Yellow River Region (YRR), 16 from the Northwestern Inland Region (NIR), six from the Yangtze River Region (YtRR), six from the Northern Special Early Maturation Region (NSEMR), and two from Southern China Region (SCR). The 27 exotic accessions were provided by the Germplasm Repository of Institute of Cotton Research, Chinese Academy of Agricultural Sciences (Anyang, Henan province, China) and were authorized for scientific research purposes only. All the accessions were inbred for at least 3 years before use in this study.

The field experiments were conducted at the Crop Research Station of Shandong Agricultural University (CRS/SDAU), Taian, China in 2014 and 2016, and in Ling County, Dezhou, China in 2015. The 196 cotton accessions were planted at two experiment sites in a randomized complete block design with three replicates. Each replicate had one row that was 8-m long. The row space was 80 cm, and the average plant space was 33 cm. The planting date was April 24 in 2014 and 2015 and April 28 in 2016. Cultural practices followed local recommendations.

### Seed fatty acid and protein determination and statistical analysis

Thirty normally opened bolls were collected from each plot at maturity stage, air-dried, and ginned with a laboratory cotton ginning machine. In each replicate, equal number of seeds of the same genotype were bulked. Then, seed coats were manually removed, and the resulting kernels were ground into powder for seed nutrients assay with three replicates. The total fatty acids (TA) (mg/g) in seed kernels and five fatty acids, including myristic acid (MA), palmitic acid (PA), stearic acid (SA), oleic acid (OA), and linoleic acid (LA), as percentages of TA were determined by gas chromatography (GC2010, Shimadzu Corporation, Kyoto, Japan) according to Lian et al. (2017). In this GC analysis, a DB-FFAP column (30 m length × 0.25 μm liquid membrane thickness × 0.32 mm inner diameter) was equipped. A total of 0.2 g seed kernel powder was loaded into a 10-mL glass tube with 2 mL of an ether-petroleum ether (1:1) solution. After mixing and shaking, the solution was left to stand overnight. Then, 2 mL of KOH in methanol (0.4 mol/L) solution and 4 mL of distilled water were added to the mixture. Next, the mixture was allowed to precipitate for 1–2 h, the supernatant and pellet were separated, and then 0.2 g of sodium sulfate anhydrous was added into the supernatant. Finally, 1 mL of the supernatant was absorbed into the GC tube for analysis. The temperature of the detector and gasification room was maintained at 250°C and 230°C, respectively, and the temperature of the column was maintained at 190°C during the first 9 min and then increased to 230°C for the next 8 min, while the flow rates of air carrier, hydrogen, and gas (nitrogen) were maintained at 400, 40, and 30 mL/min, respectively. The results were determined by the chromatographic peak area normalization method, and the mass percentage (m/%) of each component as a proportion of the total FA was calculated. The total protein (PR) content was measured using the Rapid N Exceed: N/Protein Analyzer of Elementar, Langenselbold, Germany (http://www.elementar.de/en/products/nprotein-analysis/rapid-n-exceed.html). The mean value of three replicates was used for further analysis. Phenotypic traits across multiple environments were estimated using the best linear unbiased predictions (BLUPs) based on a linear model (de et al., 2013; Huang et al., 2017a).

Statistical analysis of phenotypic data was performed using SPSS Statistics 21.0 (RRID:SCR_002865). Descriptive statistics was performed using the BLUPed traits values (Merk et al., 2012; Sun et al., 2016). The frequency distribution of each trait was calculated using R (R Core Team, Vienna, Austria).

### SNP genotyping

Genomic DNA was extracted from young leaf tissue with the Qiagen DNeasy Plant Kit. The DNA quantity and quality were measured with NanoDrop 2000 and agarose gel electrophoresis. Genotyping was conducted at the Beijing Compass Biotechnology Co., Ltd. using the CottonSNP80K array (Illumina) (Cai et al., 2017), which was developed by State Key Laboratory of Crop Genetics & Germplasm Enhancement, Hybrid Cotton R & D Engineering Research Center, Ministry of Education, Nanjing Agricultural University, Nanjing, China. Of all the 77,774 SNP loci on the array, 55,660 (71.57%) were polymorphic. Quality check for the SNP markers was performed using TASSEL v5.2.40 (RRID:SCR_012837) to remove the SNPs with a call rate of < 90% and a minor allele frequency (MAF) < 0.05. A final set of 41,815 SNP markers was retained for further analysis.

### Population structure, kinship (K), and LD analyses

The software PowerMarker version 3.25 (RRID:SCR_009332) was used to calculate the polymorphic information content (PIC) of the SNP markers, gene diversity and genetic distances among accessions, and to plot the unweighted pair group method with arithmetic means (UPGMA) phylogenetic tree using Nei's genetic distance method (Sneath and Sokal, 1973).

The population structure of the 196 accessions was estimated by STRUCTURE 2.3.4 software (Evanno et al., 2005) with the Bayesian Markov Chain Monte Carlo (MCMC) model. *K* value was set from 1 to 20, with iterations and burn-in length both set to 100,000 under the admixture and correlated allele frequencies model, and seven independent runs for each *K* were performed (Wan et al., 2017). The natural logarithms of probability data [LnP(*K*)] and the *ad hoc* statistic Δ*K* were calculated using STRUCTURE HARVESTER (http://taylor0.biology.ucla.edu/structureHarvester/), which is a program for visualizing the STRUCTURE output and implementing the Evanno method (Earl and Vonholdt, 2012; Huang et al., 2017a). The Δ*K* was considered as the determinant factor for deducing the optimal value of *K* (Mezmouk et al., 2011). Using CLUMPP software (Jakobsson and Rosenberg, 2007), the Q-matrix was obtained through integrating seven replicate runs.

Principal component analysis (PCA) and the K matrix calculated with TASSEL v5.2.40 (RRID:SCR_012837) were also used to adjust the population structure. TASSEL v5.2.40 (RRID:SCR_012837) was also used to calculate the parameter r^2^ (the correlation in frequency among pairs of alleles across a pair of SNP loci).

### Genome-wide association analysis

Genotypic and phenotypic data were jointly analyzed for determining the marker-trait associations. For this purpose, the software package TASSEL v5.2.40 (RRID:SCR_012837) was employed and the genome-wide association mapping was performed implementing six models: the naive general linear model (GLM), the general linear model considering the Q-matrix [GLM (Q)], the general linear model considering the PCA-matrix [the top six principal components, GLM (PCA)], the mixed linear model considering the K-matrix [MLM (K)], the mixed linear model considering both Q-matrix and K-matrix [MLM (Q + K)], and the mixed linear model considering both PCA-matrix and K-matrix [MLM (PCA + K)]. The threshold to define a significant association between the marker and trait was set at a probability level of –log(p) ≥ 3.8.

The LD decay distances among diverse chromosomes were set as confidence intervals for candidate-QTL regions in different chromosomes. The LD map based on the physical location was plotted using Haploview 4.2 (Calati et al., 2011). Putative candidate genes were put forward for each locus using the Cottongen JBrowse - *Gossypium hirsutum* AD1 genome NAU-NBI assembly v1.1 (annot v1.1) (https://www.cottongen.org/tools/jbrowse). Moreover, the identification of the specific expressed genes in relevant tissues of the candidate regions was based on the *G. hirsutum* (TM-1) gene expression database (Zhang et al., 2015).

## Results

### Phenotypic statistical analysis

The results of ANOVA of the traits for 3 years (2014–2016) are listed in Table 1. The results showed that there was significant (*P* < 0.01) variation among the five fatty acids (MA, myristic acid; PA, palmitic acid; SA, stearic acid; OA, oleic acid; and TA, total fatty acids) during the 3 years, whereas the observed variations in LA (linoleic acid) and PR (total protein) were not significant. The results also indicated that the environment was responsible for a sizeable portion of the observed total variations in oil and the fatty acids levels, and the interaction between the genotype and the environment for oil concentration was larger than that for the total protein content.

**Table 1:** ANOVA of seven cottonseed nutrient traits in three environments (Taian 2014, 2015 and Ling country 2016).

**Trait**	**Sum of square**			**Mean square**			***P*****-value**			**One-way ANOVA**
	**G**	**E**	**G × E**	**G**	**E**	**G × E**	**G**	**E**	**G × E**	
MA	10.588	5.298	13.966	0.054	2.649	0.036	*	*	*	0.0000
PA	2102.459	151.905	1026.511	10.782	75.952	2.632	*	*	*	0.0006
SA	199.353	25.218	150.663	1.022	12.609	0.386	*	*	*	0.0001
OA	1899.448	163.702	1270.46	9.741	81.851	3.258	*	*	*	0.0007
LA	2662.865	55.526	1534.865	13.656	27.763	3.936	*	*	*	0.1490
TA	451209.94	359334.456	346481.482	2313.897	179667.228	888.414	*	*	*	0.0000
PR	6138.913	22.532	270.019	31.482	11.266	0.692	*	*	*	0.3962

*G, genotype; E, environment; G × E, Interaction of genotype and environment*.

* *, significant at P < 0.001*.

The BLUPed phenotypic values of MA, PA, SA, OA, LA, TA, and PR followed a normal distribution (Figure S1), with mean values of 0.70%, 22.46%, 2.49%, 15.49%, 58.86%, 220.67 mg/g, and 44.27%, with the coefficient of variation (CV) of 3.99, 3.97, 8.55, 4.65, 1.58, 3.99, and 4.65%, respectively (Table 2). Correlation analysis (Table 3) found a strong negative relationship between total protein (PR) and oil (TA). Significant negative correlations were found between PA and SA and between OA and LA, whereas positive correlations were detected between MA and PA and between SA and OA. Of the five fatty acid compositions measured, only OA showed a significant negative correlation with PR, SA had significant positive correlation with PR, and the rest of the three fatty acids, namely, PA, MA, and LA, showed a very weak non-significant association with PR.

**Table 2:** Descriptive statistics of the observed phenotypic variations in seven traits.

**Trait**	**Environment**	**MA (%)**	**PA (%)**	**SA (%)**	**OA (%)**	**LA (%)**	**TA (mg/g)**	**PR (%)**
Mean	14T	0.73	22.56	2.41	15.33	58.97	241.78	44.10
	15D	0.77	21.98	2.69	16.01	58.55	199.33	44.46
	16T	0.61	22.85	2.36	15.13	59.05	209.14	44.27
	BLUP	0.70	22.46	2.49	15.49	58.86	220.67	44.27
Min	14T	0.53	19.36	1.55	13.08	54.89	202.22	37.34
	15D	0.56	18.22	1.71	13.56	53.74	165.88	37.59
	16T	0.53	20.36	1.81	13.28	56.28	173.73	37.46
	BLUP	0.62	19.86	1.88	13.78	55.99	201.90	37.71
Max	14T	0.95	29.42	4.25	18.64	61.97	286.03	48.34
	15D	1.19	28.95	4.70	21.94	63.49	259.52	48.37
	16T	0.77	27.66	3.16	18.04	61.70	242.12	48.35
	BLUP	0.81	27.77	3.49	18.62	61.46	252.76	48.21
Std. Deviation	14T	0.05	1.13	0.33	0.84	1.12	14.54	2.15
	15D	0.10	1.23	0.36	1.26	1.45	14.71	2.12
	16T	0.03	0.89	0.19	0.73	0.93	10.79	2.14
	BLUP	0.03	0.89	0.21	0.72	0.93	8.81	2.06
CV (%)	14T	7.05	4.99	13.86	5.49	1.90	6.01	4.86
	15D	12.69	5.58	13.42	7.88	2.48	7.38	4.76
	16T	5.56	3.89	8.04	4.86	1.58	5.16	4.83
	BLUP	3.99	3.97	8.55	4.65	1.58	3.99	4.65
*h*^2^	Multi-env	0.28	0.70	0.56	0.60	0.65	0.56	0.97

**Table 3:** Correlation coefficients of seven cottonseed nutrient traits.

	**MA**	**PA**	**SA**	**OA**	**LA**	**TA**
PA	0.450^**^					
SA	0.06	−0.214^**^				
OA	−0.263^**^	−0.476^**^	0.409^**^			
LA	−0.261^**^	−0.427^**^	−0.461^**^	−0.550^**^		
TA	−0.09	−0.07	−0.05	0.08	0.02	
PR	0.02	0.03	0.180^*^	−0.179^*^	0.08	−0.555^**^

*, ** *Correlation is significant at the 0.05, 0.01 level, respectively*.

### SNP genotyping and genetic diversity

**Figure 1:**
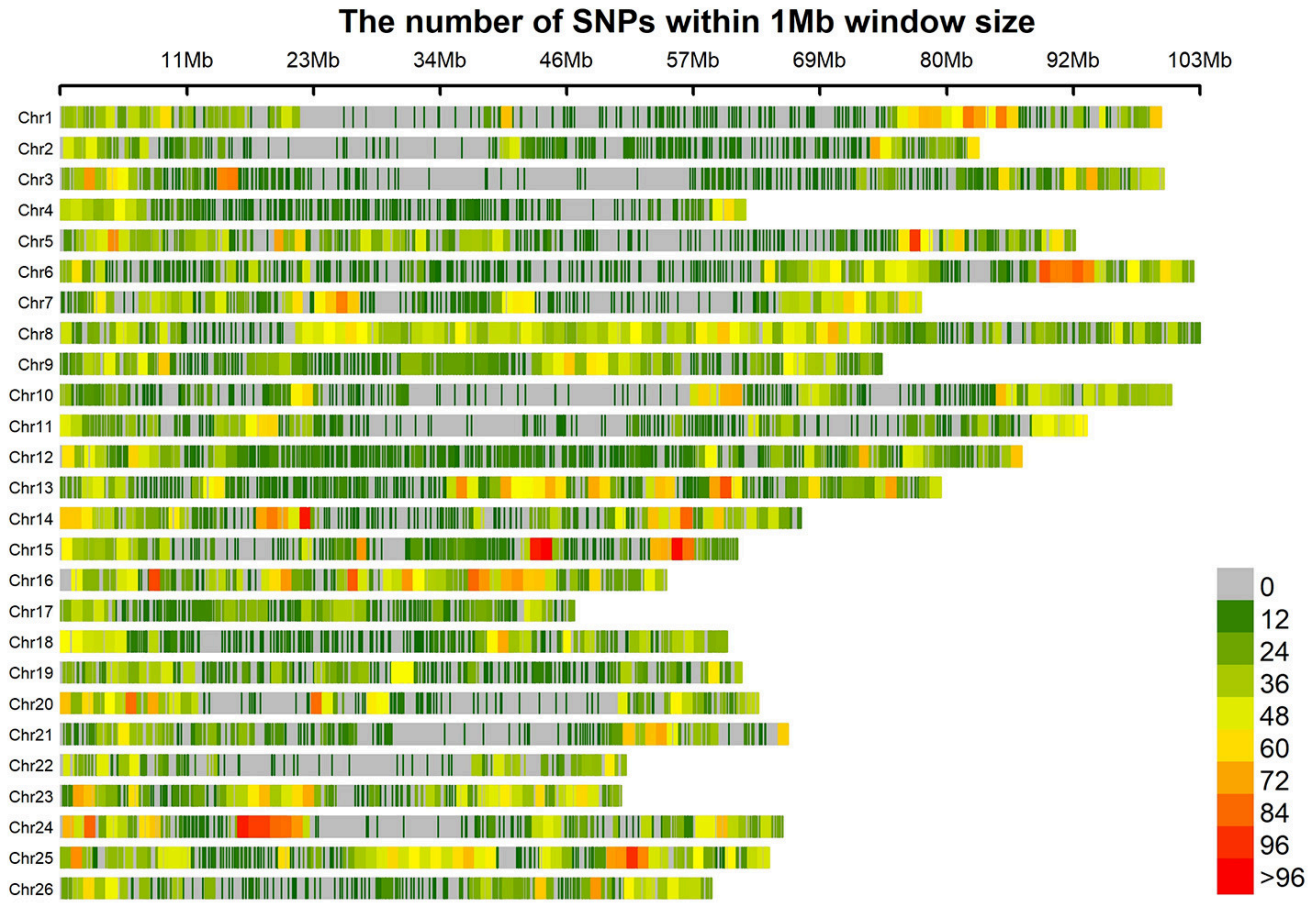
Distribution of 41815 SNPs on the 26 chromosomes of upland cotton. The horizontal axis shows chromosome length (Mb); the different colors depict SNP density (the number of SNPs per window).

All the 196 accessions were genotyped using the CottonSNP80K chip with 77,774 SNPs. Of the 77,774 SNP loci, 55,660 (71.57%) were polymorphic. After the removal of SNPs with a call rate of < 90% or with minor allele frequencies (MAFs) < 5%, 41,815 (53.76%) polymorphic SNP markers were finally screened out and used to assess the population structure (Q), relative kinship (K), and GWAS analysis. These filtered SNPs provided a whole genome-wide (1,934.65 Mb) coverage, with a mean distance of 46.26 kb (Figure 1). The average SNP distance of each chromosome ranged from 26.25 kb (Chr16) to 81.97 kb (Chr02). The PIC varied from 0.227 (Chr17) to 0.303 (Chr24), with an average of 0.267 for all SNP markers. Thus, the average gene diversity of the whole genome was 0.331, varying from 0.274 (Chr17) to 0.384 (Chr24) (Table 4).

**Table 4:** Summary of SNPs, PIC, gene diversity, and LD decay.

**Linkage group**		**Chr length**	**#SNPs**	**SNP density (Kb/SNP)**	**Gene diversity**	**PIC**	**LD decay (Mb) Rsq = 0.1**
Chr01	A01	99884.70	1880	53.130	0.368	0.293	1.90–2.00
Chr02	A02	83447.91	1018	81.972	0.339	0.272	0.45–0.55
Chr03	A03	100263.05	1380	72.654	0.350	0.280	0.40–0.50
Chr04	A04	62913.77	793	79.336	0.352	0.282	0.30–0.40
Chr05	A05	92047.02	2019	45.590	0.335	0.271	1.55–1.65
Chr06	A06	103170.44	2054	50.229	0.291	0.238	4.00–4.10
Chr07	A07	78251.02	1727	45.310	0.338	0.271	1.85–1.95
Chr08	A08	103626.34	3279	31.603	0.285	0.233	3.25–3.35
Chr09	A09	74999.93	1815	41.322	0.335	0.270	1.95–2.05
Chr10	A10	100866.60	1611	62.611	0.326	0.265	1.45–1.55
Chr11	A11	93316.19	1393	66.989	0.332	0.268	0.30–0.40
Chr12	A12	87484.87	1694	51.644	0.349	0.279	0.20–0.30
Chr13	A13	79961.12	2226	35.921	0.353	0.283	1.90–2.00
Chr14	D02	67284.55	1890	35.600	0.354	0.283	1.40–1.50
Chr15	D01	61456.01	1453	42.296	0.369	0.294	1.00–1.10
Chr16	D07	55312.61	2107	26.252	0.334	0.272	2.45–2.55
Chr17	D03	46690.66	982	47.546	0.274	0.227	5.50–5.60
Chr18	D13	60534.30	1216	49.781	0.316	0.258	1.45–1.55
Chr19	D05	61933.05	1202	51.525	0.341	0.274	0.10–0.20
Chr20	D10	63374.67	1310	48.378	0.338	0.272	0.50–0.60
Chr21	D11	66087.77	1095	60.354	0.339	0.273	0.25–0.35
Chr22	D04	51454.13	769	66.910	0.341	0.273	0.35–0.45
Chr23	D09	50995.44	1638	31.133	0.293	0.239	4.30–4.40
Chr24	D08	65894.14	1849	35.638	0.384	0.303	1.10–1.20
Chr25	D06	64294.64	2199	29.238	0.283	0.232	5.65–5.75
Chr26	D12	59109.84	1216	48.610	0.344	0.274	0.60–0.70
Total		1934654.76	41815	46.267	0.331	0.267	3.20–3.30

### LD decay, population structure, and kinship

**Figure 2:**
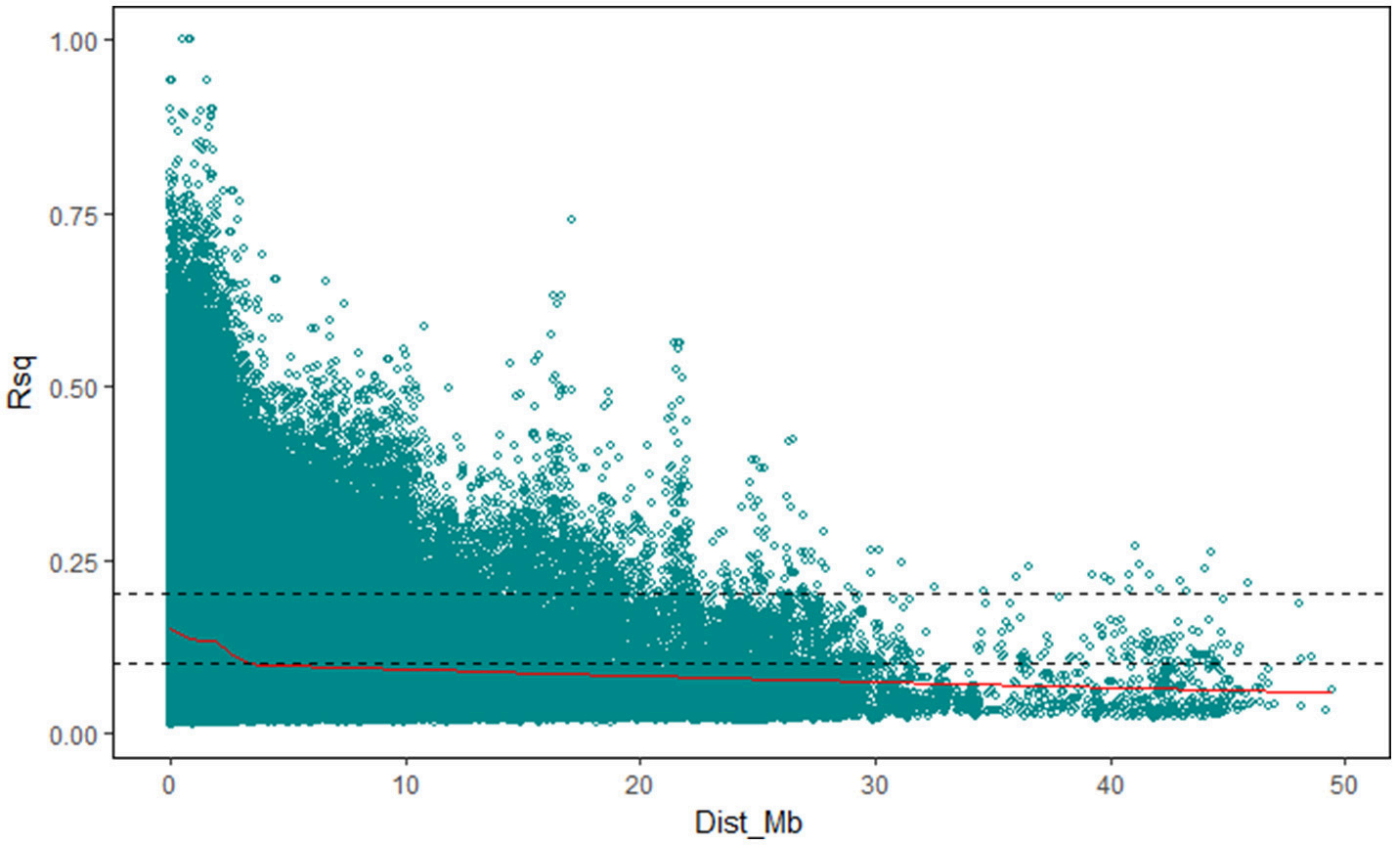
LD decay determined according to squared correlations of allele frequencies (*r*^2^).

The value of *r*^2^ between all the SNP markers genotypes in the 196 accessions, as the indicator of pairwise LD, was estimated using TASSEL v5.2.40. In this study, the average *r*^2^ at each 0.1 Mb was set as a function for inter-marker distance and used to estimate the LD decay in the upland cotton population, and the cut-off value of *r*^2^ was set to 0.1. The LD decay distance in the 196 accessions among all the SNP markers was 3.20–3.30 Mb (Figure 2). Furthermore, the LD decays occurred unevenly among different chromosomes (Table 4; Figure S2), ranging from 0.10–0.20 Mb (Chr19) to 5.65–5.75 Mb (Chr25). The Dt-subgenome chromosomes showed a smaller LD decay range than the At-subgenome chromosomes. Similar LD decays were detected between homoeologous chromosomes, such as Chr04-Chr22 (0.30–0.45 Mb) and Chr11-Chr21 (0.25–0.40 Mb), whereas distinct LD decay differences (>1.45 Mb) were observed in some other homologous chromosomes, e.g., Chr03-Chr17, Chr05-Chr19, Chr06-Chr25, Chr08-Chr24, and Chr09-Chr23.

**Figure 3:**
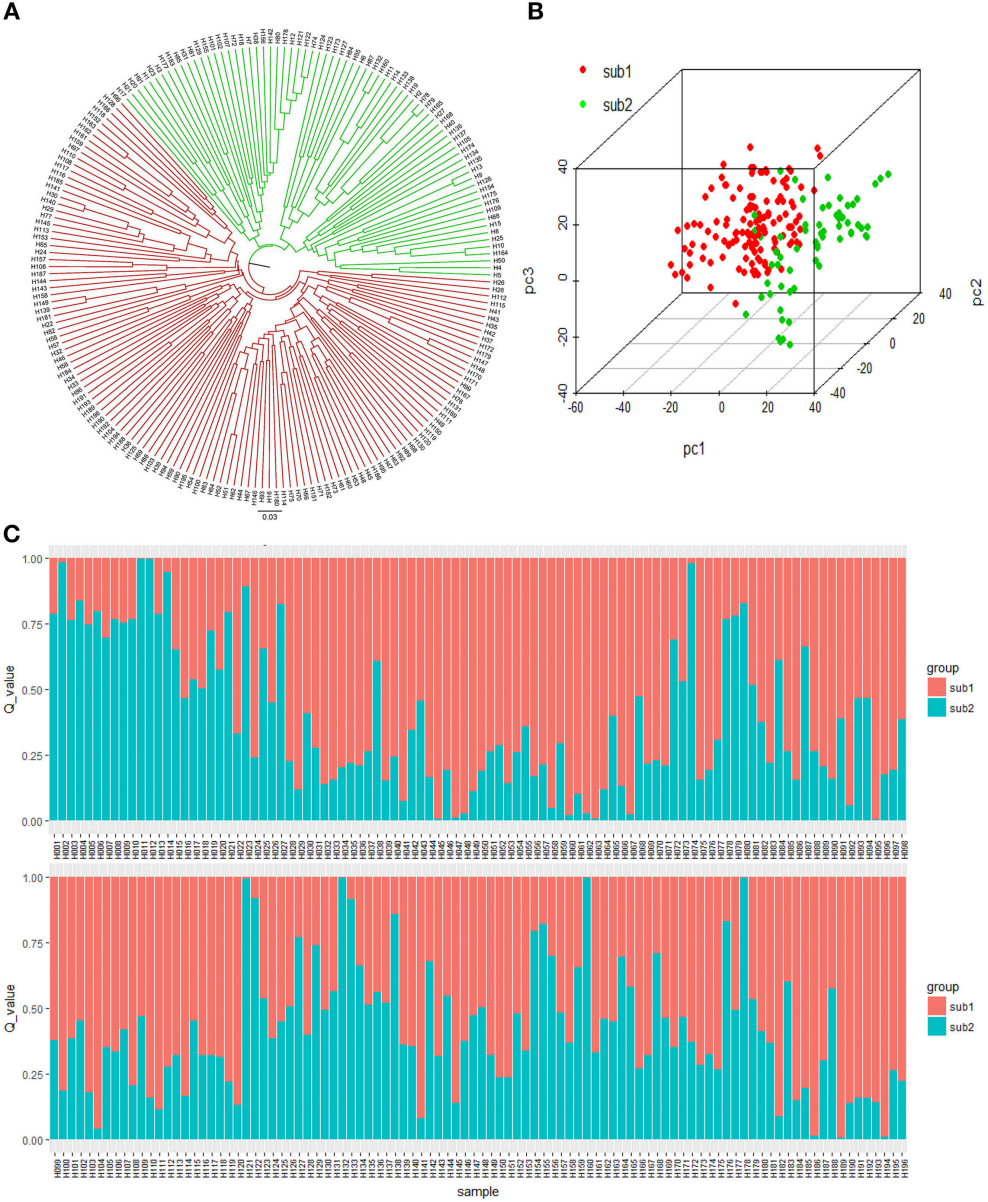
Population structure of the 196 accessions. **(A)** UPGMA tree based on Nei's genetic distances. **(B)** Principal component analysis of 196 accessions based on genotype. **(C)** Population structure of the 196 accessions based on STRUCTURE when *K* = 2.

Three methods were adopted to estimate the number of subpopulations of the 196 accessions basing the genotypic data, seeing that population structure affects the authenticity of QTL mapping. First, the UPGMA phylogeny tree based on Nei's genetic distances grouped 196 accessions into two major clusters (Figure 3A). Second, the Bayesian clustering was performed for *K* = 1–20 with seven repetitions using the software STRUCTURE. There was no an obvious inflection point in the curve of LnP(D)~K (from *K* = 1 to *K* = 20) (Figure S3A). However, a distinct spike value of the Evanno's DK was shown at *K* = 2 (Figure S3B), suggesting that the population could be divided into two subgroups (Figure 3C). Third, the genotypic PCA showed that the front three eigenvectors occupied only 15.84% of the observed genetic variations, with PC1, PC2, and PC3 accounting for 7.45, 4.53, and 3.85%, respectively. The PCA spatial distribution map showed that the population was divided into two subgroups with few overlapping regions (Figure 3B). The K matrix, another important factor for GWAS, was visualized using a heatmap (Figure S4), in which the two subpopulations were clearly separated. Overall, the results of the phylogeny tree, structure, PCA, and K matrix proved that the 196 accessions consisted of two subpopulations, containing 133 genotypes (Sub1) and 63 genotypes (Sub2), respectively (Table S1).

### Marker-trait associations

**Figure 4:**
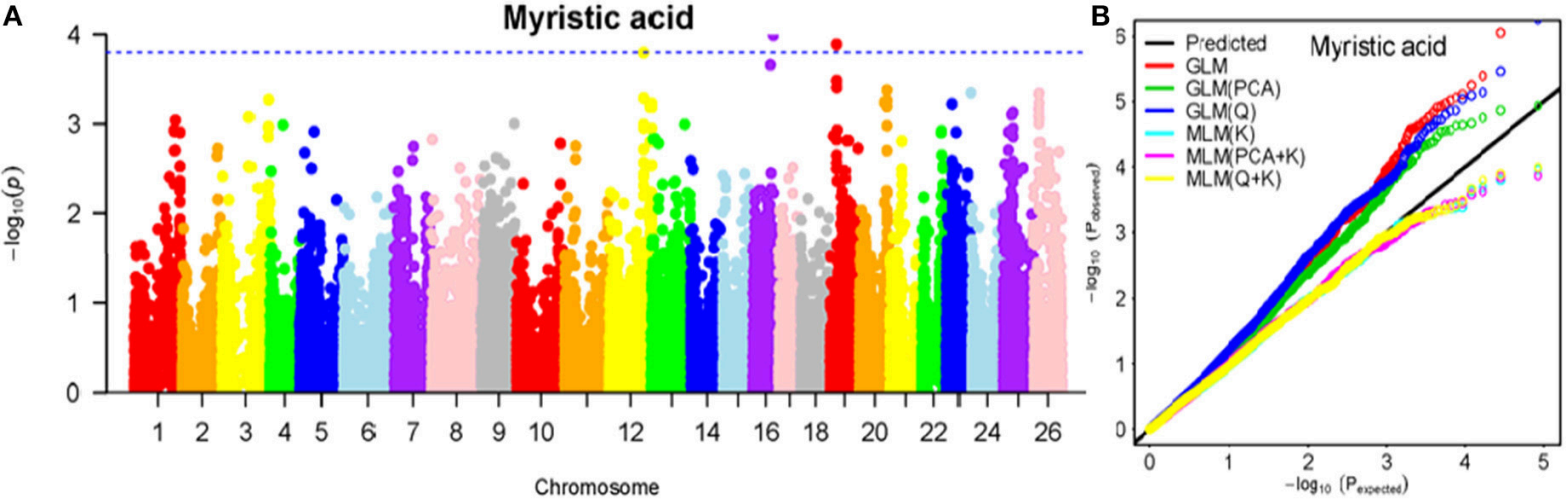
Summary of GWAS results for MA. **(A)** Manhattan plot for MA GWAS results. The threshold value was set at –log(p) > 3.80. **(B)** Q-Q plots for FP using GLM, GLM (Q), GLM (PCA), MLM (K), MLM (PCA+K), and MLM (Q+K).

**Figure 5:**
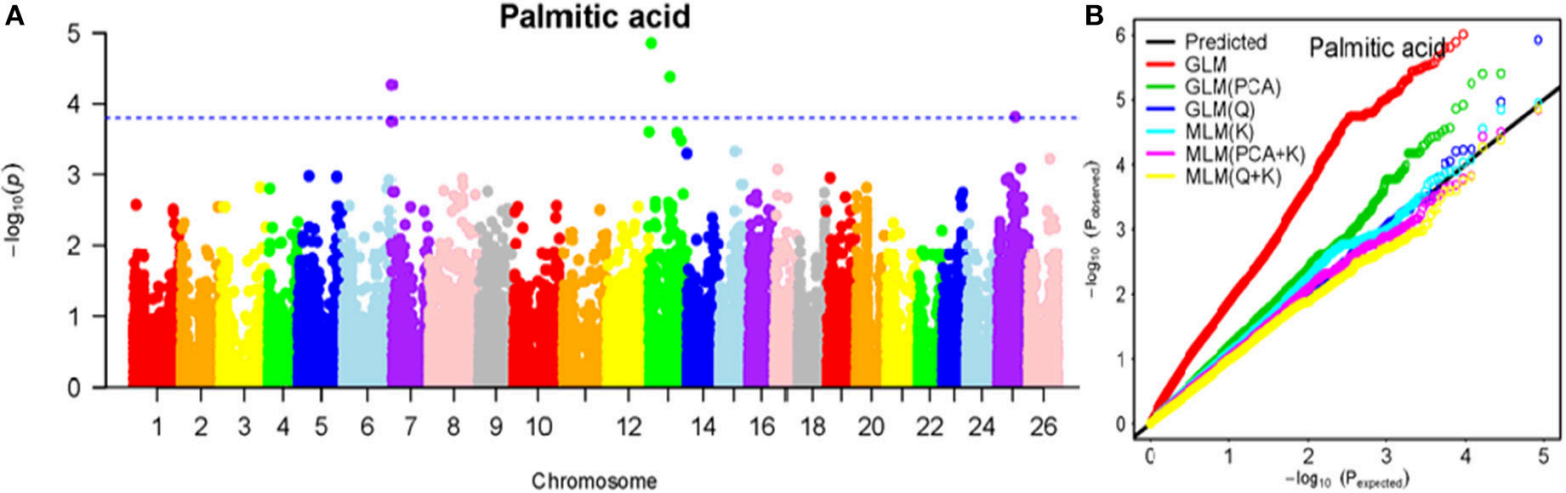
Summary of GWAS results for PA. **(A)** Manhattan plot for PA GWAS results. The threshold value was set at –log(p) > 3.80. **(B)** Q-Q plots for FP using GLM, GLM (Q), GLM (PCA), MLM (K), MLM (PCA+K), and MLM (Q+K).

**Figure 6:**
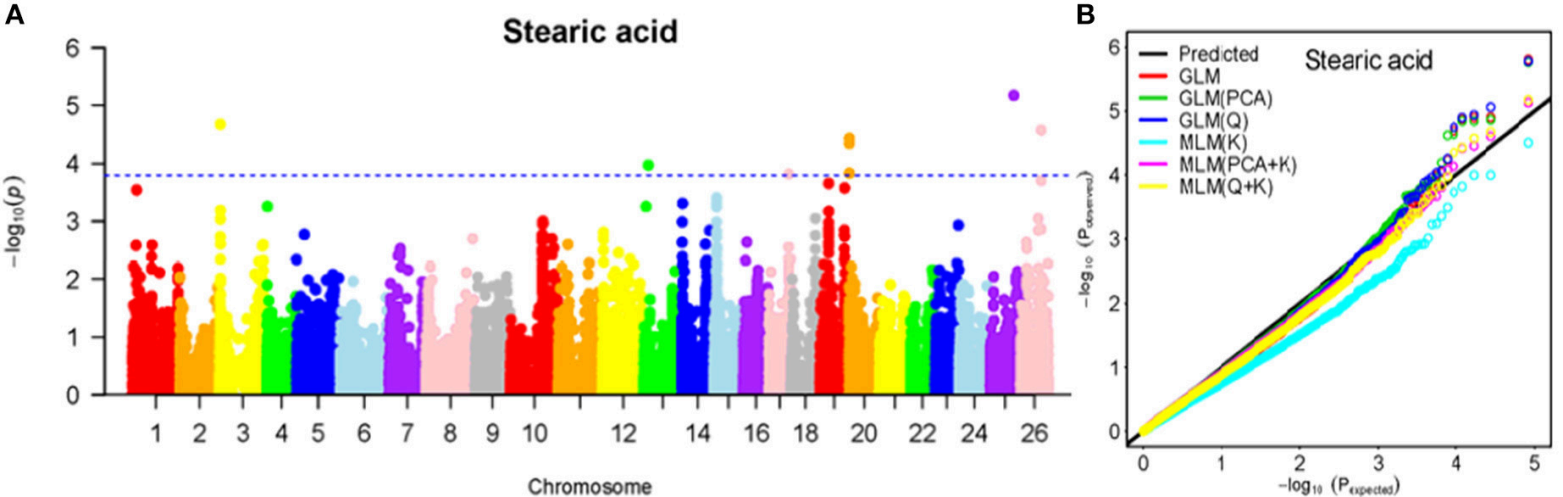
Summary of GWAS results for SA. **(A)** Manhattan plot for SA GWAS results. The threshold value was set at –log(p) > 3.80. **(B)** Q-Q plots for FP using GLM, GLM (Q), GLM (PCA), MLM (K), MLM (PCA+K), and MLM (Q+K).

**Figure 7:**
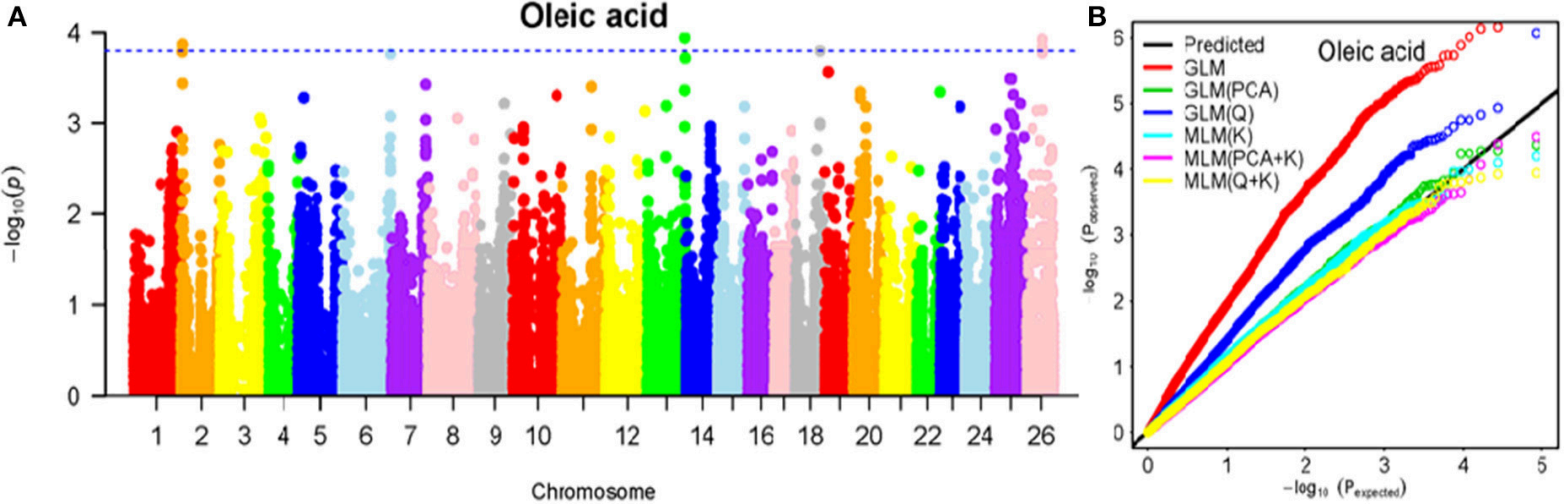
Summary of GWAS results for OA. **(A)** Manhattan plot for OA GWAS results. The threshold value was set at –log(p) > 3.80. **(B)** Q-Q plots for FP using GLM, GLM (Q), GLM (PCA), MLM (K), MLM (PCA+K), and MLM (Q+K).

**Figure 8:**
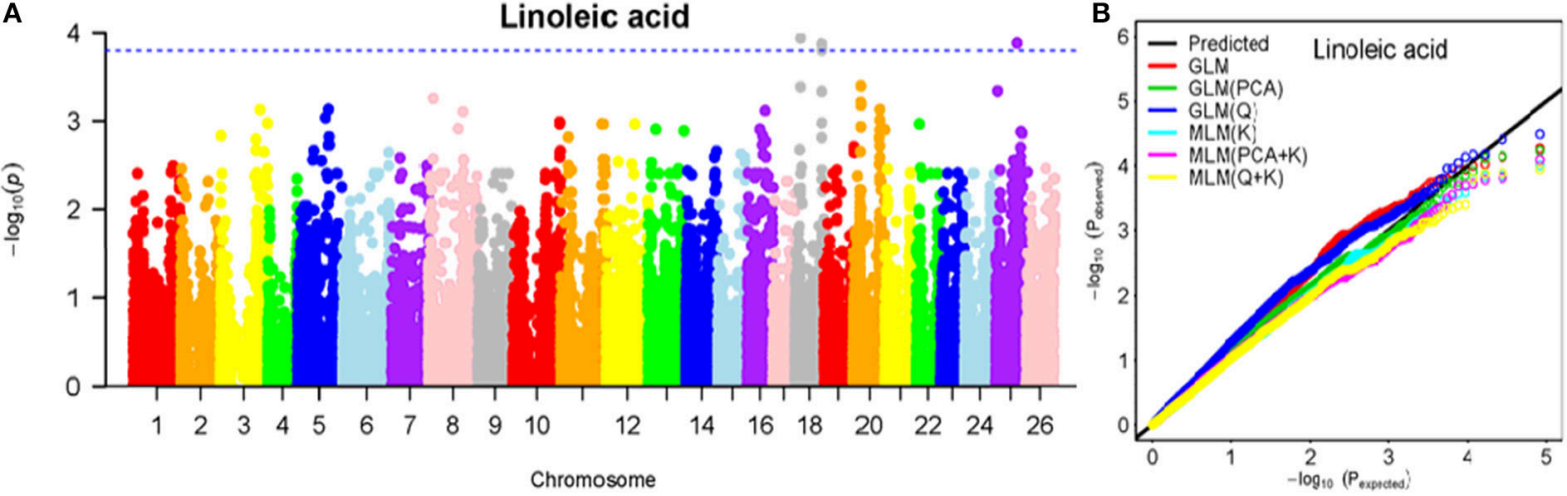
Summary of GWAS results for LA. **(A)** Manhattan plot for TA GWAS results. The threshold value was set at –log(p) > 3.80. **(B)** Q-Q plots for FP using GLM, GLM (Q), GLM (PCA), MLM (K), MLM (PCA+K), and MLM (Q+K).

**Figure 9:**
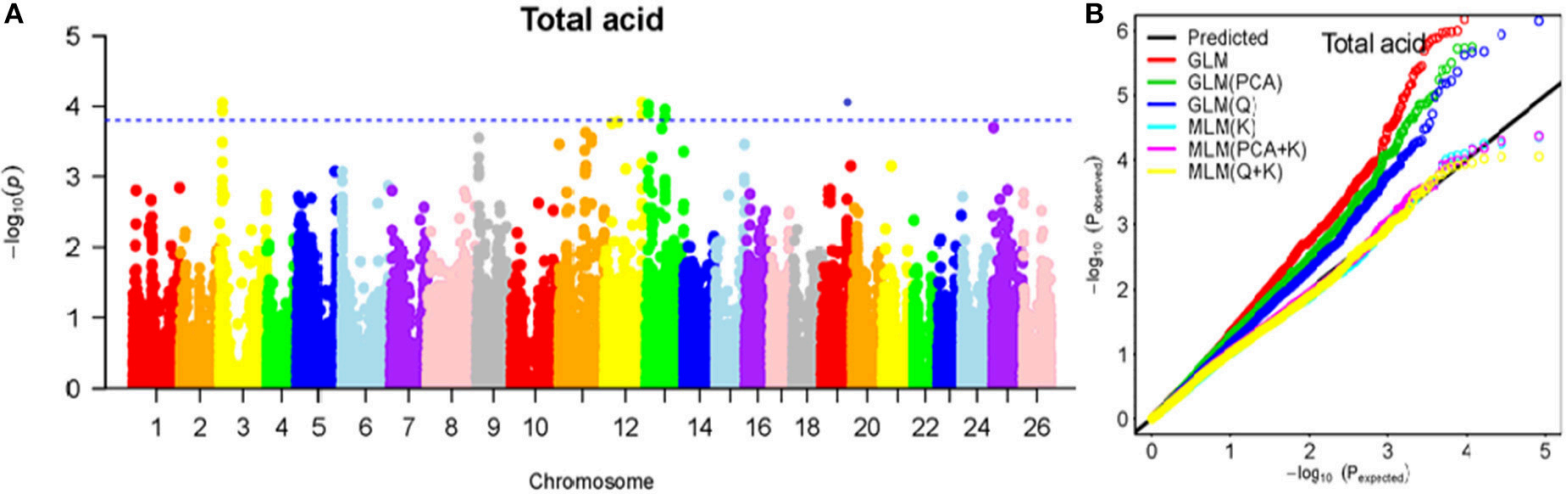
Summary of GWAS results for TA. **(A)** Manhattan plot for LA GWAS results. The threshold value was set at –log(p) > 3.80. **(B)** Q-Q plots for FP using GLM, GLM (Q), GLM (PCA), MLM (K), MLM (PCA+K), and MLM (Q+K).

**Figure 10:**
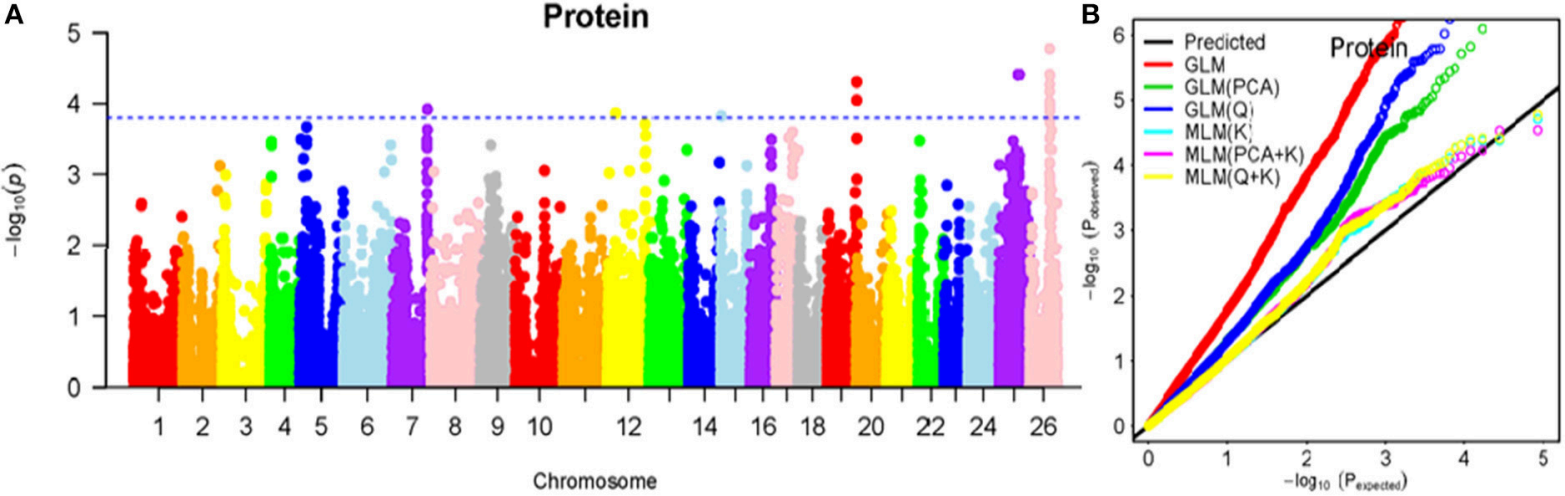
Summary of GWAS results for PR. **(A)** Manhattan plot for PR GWAS results. The threshold value was set at –log(p) > 3.80. **(B)** Q-Q plots for FP using GLM, GLM (Q), GLM (PCA), MLM (K), MLM (PCA+K), and MLM (Q+K).

To determine the most appropriate model for association analysis, six common models, namely, the GLM model, the GLM (Q) model, the GLM(PCA) model, the MLM (K) model, the MLM (PCA + K) model, and the MLM (Q + K) model, were compared and shown using a quantile-quantile (Q-Q) plot (Figures 4B–10B). In the Q–Q plot of the seven seed nutrients, the scatter-lines based on the naive GLM, Q models, and PCA models clearly deviated from expectation, and hence the mixed linear models (MLM (K), MLM (PCA + K), and MLM (Q + K)) performed significantly better than the general linear models (naive GLM, GLM+Q, and GLM+PCA). The mixed linear model [MLM (K)] only reduced the errors in K compared to the MLM (Q + K) and MLM (PCA + K) models, which controlled both population structure (Q) and kinship (K). To make the most efficient use of the phenotypic and genotypic data in this study, the MLM (Q + K) model was finally selected for GWAS analysis, considering that the top two eigenvectors of PCA only accounted for 11.98% of the observed genetic variation.

The BLUPed traits and 41,815 SNP markers were used in the association analysis. With the Q+K model, 47 significantly associated SNP markers (–log(p) > 3.80) were identified for seven cottonseed nutrient traits (Table S2, Table 5; Figures 4A–10A). In addition, 40, 44, 39, 36, and 30 of the 47 SNP markers were verified in the GLM, GLM (Q), GLM (PCA), MLM (K), and MLM (PCA + K) models, respectively, suggesting the repeatability and reliability of the MLM (Q + K) model (Table S2). Furthermore, most of the significant SNP markers associated with the BLUPed traits were also detected in a single environment (Table 6), thereby suggesting the reliability of the marker-BLUPed trait associations. Among the 47 significant SNP markers, 2, 4, 8, 4, 4, 8, and 17 SNP markers were associated with MA, OA, SA, PA, LA, TA, and PR, respectively. The phenotypic variation explained by these SNP markers (R^2^) ranged from 7.3 to 13.3%, with an average of 9.5%.

**Table 5:** Significant SNPs, QTLs, and candidate genes associated with seven cottonseed nutrient traits.

**Trait**	**Marker**	**Chr**	**Pos(bp)**	**–lg(*P*)**	**Marker R^2^(%)**	**QTL Name**	**Physical Chr**	**Genome position range (bp)**	**Region (Mb)**	**No. of sign -ificant SNP**	**Max (-lg*P*)**	**Max *R*^2^(%)**	**No. of genes**
LA	TM80412	Chr18	7,543,336	3.943	10.45	*qGhLA-c18-1*	D13	6493239.7787560	1.294	1	3.94	10.45	59
LA	TM81777	Chr18	52,219,361	3.804	9.5	*qGhLA-c18-2*	D13	52119700.52251562	0.132	2	3.88	9.52	9
LA	TM81779	Chr18	52,235,958	3.876	7.84								
LA	TM61555	Chr25	41,801,658	3.886	10.11	*qGhLA-c25*	D06	39173974.45663184	6.489	1	3.89	10.11	119
MA	TM66249	Chr16	44,950,059	3.991	10.64	*qGhMA-c16*	D07	44946097.45467193	0.521	1	3.99	10.64	15
MA	TM57305	Chr19	15,335,570	3.889	9.70	*qGhMA-c19*	D05	15246998.16560944	1.314	1	3.89	9.70	125
OA	TM3960	Chr02	3,986,589	3.870	9.94	*qGhOA-c2*	A02	3961177.4224612	0.263	1	3.87	9.94	20
OA	TM47640	Chr13	77,932,236	3.943	7.96	*qGhOA-c13*	A13	77912110.78678331	0.766	1	3.94	7.96	73
OA	TM78520	Chr26	34,768,467	3.833	7.5	*qGhOA-c26*	D12	34068467.35548138	1.480	2	3.92	7.64	66
OA	TM78525	Chr26	34,848,138	3.924	7.64								
PA	TM18505	Chr07	710,669	4.269	8.20	*qGhPA-c7*	A07	293465.714551	0.421	1	4.27	8.20	29
PA	TM43451	Chr13	5,406,222	4.857	10.16	*qGhPA-c13-1*	A13	3406222.7406222	4.000	1	4.86	10.16	171
PA	TM45415	Chr13	44,886,205	4.381	10.34	*qGhPA-c13-2*	A13	44760913.44965389	0.204	1	4.38	10.34	1
PA	TM61185	Chr25	36,901,571	3.815	7.29	*qGhPA-c25*	D06	31151571.42651571	11.500	1	3.82	7.29	157
PR	TM21332	Chr07	72,338,466	3.921	7.97	*qGhPR-c7*	A07	70388466.74288466	3.900	1	3.92	7.97	161
PR	TM40785	Chr12	15,670,185	3.866	9.74	*qGhPR-c12*	A12	15568074.15792258	0.224	1	3.87	9.74	7
PR	TM47774	Chr15	746,934	3.827	7.78	*qGhPR-c15*	D01	738305.771729	0.033	1	3.83	7.78	1
PR	TM58723	Chr19	59,003,770	4.307	10.8	*qGhPR-c19*	D05	58997573.59014635	0.017	2	4.31	10.82	1
PR	TM58736	Chr19	59,093,748	4.047	10.21								
PR	TM61525	Chr25	39,635,535	4.410	11.32	*qGhPR-c25*	D06	39173974.44145093	4.971	1	4.41	11.32	103
PR	TM78699	Chr26	38,662,762	3.987	8.14	*qGhPR-c26*	D12	38644336.40094524	1.450	11	4.78	12.50	63
PR	TM78714	Chr26	38,746,510	3.893	9.78								
PR	TM78716	Chr26	38,752,485	3.951	9.97								
PR	TM78728	Chr26	38,818,102	4.173	10.84								
PR	TM78729	Chr26	38,824,393	4.410	11.50								
PR	TM78734	Chr26	38,854,900	4.777	12.50								
PR	TM78739	Chr26	38,878,451	3.917	10.23								
PR	TM78747	Chr26	38,927,683	4.023	10.23								
PR	TM78755	Chr26	38,977,054	3.889	9.77								
PR	TM78763	Chr26	39,024,108	4.272	8.89								
PR	TM78772	Chr26	39,394,524	4.396	9.19								
SA	TM5908	Chr03	2,256,335	4.672	9.81	*qGhSA-c3*	A03	2020160.2281287	0.261	1	4.67	9.81	17
SA	TM43574	Chr13	10,045,412	3.975	8.17	*qGhSA-c13*	A13	8045412.12045412	4.000	1	3.97	8.17	71
SA	TM55186	Chr17	42,621,423	3.818	7.78	*qGhSA-c17*	D03	42616598.48221423	5.605	1	3.82	7.78	274
SA	TM73133	Chr20	1,415,905	3.833	7.0	*qGhSA-c20*	D10	815905.2180586	1.365	3	4.43	11.42	153
SA	TM73142	Chr20	1,492,674	4.350	10.91								
SA	TM73143	Chr20	1,495,902	4.427	11.42								
SA	TM62122	Chr25	51,051,243	5.173	13.33	*qGhSA-c25*	D06	50971548.51381533	0.410	1	5.17	13.33	10
SA	TM79132	Chr26	44,288,210	4.570	9.61	*qGhSA-c26*	D12	43588210.44988210	1.400	1	4.57	9.61	63
TA	TM6258	Chr03	6,104,050	3.936	8.2	*qGhTA-c3*	A03	6080750.6121903	0.041	2	4.05	8.37	1
TA	TM6260	Chr03	6,121,903	4.048	8.37								
TA	TM42817	Chr12	78,890,369	4.052	8.2	*qGhTA-c12*	A12	78878742.78975105	0.096	2	4.05	9.78	7
TA	TM42822	Chr12	78,934,096	3.889	9.78								
TA	TM43434	Chr13	5,306,522	3.910	10.3	*qGhTA-c13-1*	A13	5304414.5486302	0.182	2	4.02	10.34	3
TA	TM43444	Chr13	5,364,845	4.018	8.77								
TA	TM44855	Chr13	40,573,896	3.845	8.2	*qGhTA-c13-2*	A13	40377247.40698806	0.322	2	3.95	8.73	10
TA	TM44865	Chr13	40,639,827	3.955	8.73								

**Table 6:** Significant SNP markers associated with BLUPed traits shared with those markers detected in a single environment.

**Trait**	**Marker**	**Chr**	**Pos(bp)**	**Environments**	**–lg(*P*)**	**Marker *R*^2^(%)**
LA	TM80412	Chr18	7,543,336	BLUP,16T	3.84–3.94	9.95–10.45
LA	TM81777	Chr18	52,219,361	BLUP,15D	3.80–4.61	9.52–11.68
LA	TM81779	Chr18	52,235,958	BLUP,15D	3.88–4.51	7.84–9.39
LA	TM61555	Chr25	41,801,658	BLUP,15D	3.89–4.28	10.11–11.14
MA	TM66249	Chr16	44,950,059	BLUP,15D	3.99–4.86	10.64–13.24
MA	TM57305	Chr19	15,335,570	BLUP,15D	3.89–4.43	9.7–11.15
OA	TM3960	Chr02	3,986,589	BLUP,15D	3.87–4.12	9.94–10.75
OA	TM47640	Chr13	77,932,236	BLUP,14T	3.94–3.98	7.96–8.18
OA	TM78520	Chr26	34,768,467	BLUP	3.83	7.52
OA	TM78525	Chr26	34,848,138	BLUP	3.92	7.64
PA	TM18505	Chr07	710,669	BLUP,15D,16T	4.16–5.02	7.99–9.75
PA	TM43451	Chr13	5,406,222	BLUP,14T,15D,16T	4.23–4.86	8.5710.16
PA	TM45415	Chr13	44,886,205	BLUP,14T,16T	3.85–5.38	8.86–13.11
PA	TM61185	Chr25	36,901,571	BLUP	3.82	7.29
PR	TM21332	Chr07	72,338,466	BLUP,15D,16T	3.92–4.02	7.97–8.22
PR	TM40785	Chr12	15,670,185	BLUP,14T,15D,16T	3.82–3.87	9.60–9.74
PR	TM47774	Chr15	746,934	BLUP,15D,16T	3.83–3.94	7.78–8.06
PR	TM58723	Chr19	59,003,770	BLUP,14T,15D,16T	4.22–4.33	10.60–10.84
PR	TM58736	Chr19	59,093,748	BLUP,14T,15D,16T	3.84–4.19	9.66–10.59
PR	TM61525	Chr25	39,635,535	BLUP,14T,15D,16T	4.19–4.50	10.73–11.55
PR	TM78699	Chr26	38,662,762	BLUP,14T,15D,16T	3.87–4.06	7.86–8.32
PR	TM78714	Chr26	38,746,510	BLUP,15D,16T	3.89–4.05	9.78–10.21
PR	TM78716	Chr26	38,752,485	BLUP,15D,16T	3.95–4.09	9.97–10.34
PR	TM78728	Chr26	38,818,102	BLUP,14T,15D,16T	3.95–4.34	10.19–11.33
PR	TM78729	Chr26	38,824,393	BLUP,14T,15D,16T	4.32–4.43	11.25–11.56
PR	TM78734	Chr26	38,854,900	BLUP,14T,15D,16T	4.64–4.85	12.08–12.72
PR	TM78739	Chr26	38,878,451	BLUP,14T,15D,16T	3.80–3.97	9.87–10.41
PR	TM78747	Chr26	38,927,683	BLUP,14T,15D,16T	3.83–4.16	9.72–10.57
PR	TM78755	Chr26	38,977,054	BLUP,15D,16T	3.89–4.04	9.77–10.19
PR	TM78763	Chr26	39,024,108	BLUP,14T,15D,16T	4.15–4.32	8.62–8.99
PR	TM78772	Chr26	39,394,524	BLUP,14T,15D,16T	4.19–4.55	8.67–9.59
SA	TM5908	Chr03	2,256,335	BLUP,14T	3.87–4.67	9.24–9.81
SA	TM43574	Chr13	10,045,412	BLUP,15D	3.98–6.08	8.17–13.50
SA	TM55186	Chr17	42,621,423	BLUP,15D	3.82–4.72	7.78–9.99
SA	TM73133	Chr20	1,415,905	BLUP,15D	3.83–4.37	7.74–9.07
SA	TM73142	Chr20	1,492,674	BLUP,15D	4.35–5.16	10.91–13.08
SA	TM73143	Chr20	1,495,902	BLUP,15D	4.427–5.37	11.42–13.92
SA	TM62122	Chr25	51,051,243	BLUP,14T	5.17–5.87	13.33–17.95
SA	TM79132	Chr26	44,288,210	BLUP,15D	4.57–5.79	9.61–12.72
TA	TM6258	Chr03	6,104,050	BLUP,15D	3.94–4.12	8.21–8.58
TA	TM6260	Chr03	6,121,903	BLUP,15D	4.05–4.25	8.78
TA	TM42817	Chr12	78,890,369	BLUP,14T,16T	3.89–4.38	7.83–9.08
TA	TM42822	Chr12	78,934,096	BLUP,16T	3.85–3.89	9.67–9.78
TA	TM43434	Chr13	5,306,522	BLUP,14T	3.91–4.08	10.34–10.90
TA	TM43444	Chr13	5,364,845	BLUP,14T	4.02–4.11	8.77–9.11
TA	TM44855	Chr13	40,573,896	BLUP,16T	3.85–4.54	8.22–10.15
TA	TM44865	Chr13	40,639,827	BLUP,16T	3.96–4.37	8.73–9.67

If the distance between the lead SNP and following SNP markers was less than the LD decay distance among each chromosome or the pairwise r2 (the LD statistic) between the lead SNP and the following SNP markers was >0.1, then these SNP markers were set as a confidence interval for a QTL. Consequently, from the 47 associated SNP markers (Table 5; Table S3), 28 QTLs were identified on 13 chromosomes, including four pairs of homologous chromosomes (A03-D03, A07-D07, A12-D12, and A13-D13) containing 19 QTLs (67.9%). For PR, six QTLs were located on six chromosomes, explaining 7.78–12.50% of the observed phenotypic variation (PV). Four QTLs conferring TA, located on three chromosomes (A03, A12, and A13) explained 8.37–10.34% of the PV. As for fatty acid composition, six QTLs for SA were mapped to six chromosomes and accounted for 7.78–13.33% of the PV. The number of QTLs controlling LA, MA, OA, and PA was 3, 2, 3, and 4, and the PV explained was 9.52–10.45%, 9.70–10.64%, 7.64–9.94%, and 7.29–10.34%, respectively (Table 5; Table S3).

The number of significant SNP markers within the QTL regions varied from 1 to 11. Nine QTL regions contained two or more significant SNP markers, of which the *qGhPR-c26* region contained the maximum of 11 significant SNP markers (Table 5). According to the physical positions of SNP markers intervals for QTLs, the 28 QTLs were assigned to physical regions on the *G. hirsutum* (TM-1) genome, and a total of 1,789 genes were localized to these riveted regions (Table 5). Each QTL region contained 1 to 274 genes, with an average of 64 genes.

### Candidate gene approach

**Figure 11:**
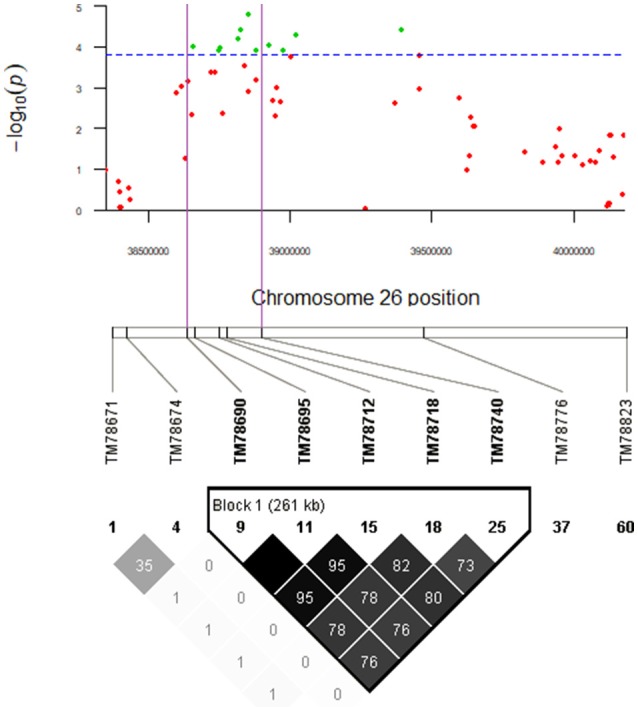
*qGhPR-c26* on Chr26 (D12: 38644336..40094524) was associated with protein content in seed. Manhattan plot shown for the D12: 38644336..40094524 region. The green plot in purple region of Manhattan plot and the bold SNP markers in Block 1 (261kb) were significantly associated with PR.

There was only one QTL, *qGhPR-c26*, which encompassed more than five significant SNPs. Thus, the LD decay distance of that QTL was narrow and less than 1 Mb at *r*^2^ < 0.1. The *qGhPR-c26* accounted for 10.09% of the PR (total protein) variation, and the minimum *P*-value was 0.0000167. Combining the QTL *qGhPR-c26* (D12:338644336..0094524) and the LD heatmap (TM78688_TM78690 – TM78740_TM78747: D12:38600352..38927683) (Figure 11), the candidate region was further narrowed down to the region D12:38644336..3892683, and eight genes were located in this region (Table S4). All the candidate genes of fatty acid and total protein were already annotated in *A. thaliana* (Table S5). Of these, Gh_D12G1162 and Gh_D12G1165 were preferentially expressed in the ovules at 5, 10, and 20 DPA, and Gh_D12G1161 was preferentially expressed in the ovules at 20, 25, and 35 DPA, based on the TM-1 gene expression database (Tables S4, S5).

According to the TM-1 gene expression database (Zhang et al., 2015), a number of genes located in the QTL confidence intervals in the present study had specific temporal and spatial expression patterns in the roots, stems, leaves, and ovules (5 DPA, 10 DPA, 20 DPA, 25 DPA, and 35 DPA). Therefore, these genes are considered to be involved in the corresponding features. For example, the genes particularly or preferentially expressed in the ovule may be associated with seed oil content and protein content. After screening, 89 genes preferentially expressed in the ovules were found and were considered as the potential candidate genes for 21 QTLs (Table S6). The number of genes located in one QTL region ranged from 5 for TA to 26 for SA.

The annotation information of *A. thaliana* was also used as a reference for the screening of candidate genes (Table S7). Several genes coding key enzymes in fatty acid synthesis were screened out from the fatty acid-related QTL regions. For example, Gh_D13G1748, located in *qGhLA-c18-2* (Table S8), was annotated to encode the acyl carrier protein 5, which modulates the fatty acid composition in *A. thaliana* (Huang et al., 2017b). SA is the substrate for the synthesis of OA. The gene, Gh_D12G1429, located in *qGhSA-c26*, was annotated to encode fatty acyl-ACP thioesterase B (FATB), which enhances the quality of cottonseed oil with high OA (Liu et al., 2017a). The β-ketoacyl-acyl carrier protein synthase III (KAS III) is one of the main factors affecting the initiation step of the fatty acid chain, involving a Claisen condensation of the acetyl-CoA starter unit with the first extender unit, malonyl-ACP, to form acetoacetyl-ACP (Dawe et al., 2003; Abugrain et al., 2017). The *qGhSA-c17* region contained a KAS III gene, Gh_D03G1548, implying its involvement in fatty acid synthesis.

## Discussion

### Correlation and simultaneous improvement of cottonseed protein and oil

Cottonseed, an important by-product of cotton, is produced in large amounts every year around the world, and has been used in solving health and starvation problems caused by the increasing world population (Cai et al., 2010; Liu et al., 2017b). The improvement of cottonseed through breeding has been gaining increasing attention in the recent years. However, the well documented negative correlation between cottonseed protein and oil and the complex genetic control hinder their simultaneous improvement in the conventional cotton breeding programs (Song and Zhang, 2007; Yu et al., 2012; Badigannavar and Myers, 2015; Liu et al., 2015a,b). We also detected such negative correlation between cottonseed protein and oil and found that of the five fatty acid compositions measured, only OA is negatively correlated to PR. The other four fatty acids, namely, SA, PA, LA, and MA, showed significant positive or very weak non-significant correlation with PR (Table 3). These findings suggest that the TA-PR negative correlation might be due to, at least to some extent, the OA-PR negative correlation in this panel. These results implied the possibility to increase PR together with SA, PA, LA, and MA by maintaining the OA level in breeding. The strong, negative relationship between oil and protein could be because of the linked QTLs/SNP markers that separately regulate their concentrations or due to the pleiotropic effects of some QTLs/SNP markers (Chung et al., 2003). In the present study, we detected qGhLA-c25 with the additive effect to increase LA, and qGhPR-c25 having an additive effect to reduce PR, within the same region on Chr25. Thus, this region is difficult to use in improving LA and PR simultaneously.

### Precision in GWAS

GWAS is an alternative approach that makes use of a number of recombination events that have occurred within the evolutionary history of natural populations, circumventing the limitations of linkage map analysis (Rafalski, 2010). GWAS has been widely used to detect QTLs and to dissect the genetic architecture of complex quantitative traits in plants (Edwards et al., 2013; Saïdou et al., 2014). Population genetic diversity levels, phenotyping accuracy, marker density, and statistical algorithms are the major factors influencing the power of GWAS.

Therefore, an association population should cover a large number of accessions to encompass the genetic diversity as much as possible. However, working with large populations may be prone to errors because of the differences in field environments and management measures, especially for the cotton being a large-plant crop, which could decrease the detection accuracy of association analysis (Liu et al., 2015b). Hence, core or mini-core collection is a useful choice. In addition, adding exotic germplasm to increase the level of geographical distribution and phenotypic variation of the population is a common practice. The upland cotton panel was a sub collection from 274 accessions and covered a large geographical distribution, with a relatively high average PIC of 0.267 and a high phenotypic variation (Table 2).

For cottonseed oil and protein content, both additive and non-additive (including dominance) effects have been reported (Kohel, 1980; Dani and Kohel, 1989; Wu et al., 2009, 2010). Recently, significant epistatic effects on the oil and protein content were also detected in the study of Du et al. (2018). They also detected a significant interaction effect between epistasis and environment only for the oil content. In our study, the environment was also responsible for a sizeable portion of the observed total variations in oil and protein content, and the interaction between the genotype and the environment in oil concentration was larger than that for total protein content. Due to strong genotype × environment interactions in complex quantitative traits, phenotyping under multi-environments is usually adopted to eliminate/minimize the environment effect in GWAS. Despite such an approach, a significant proportion of the genetic variation is still unaccounted for, and the accuracy of prediction is usually low. Evidence shows that the accuracy of prediction can be improved when the phenotypes are regressed on hundreds of thousands of variants simultaneously using whole-genome regression (WGR) models (de et al., 2013). The BLUP, which is a commonly adopted WGR method with high prediction accuracy in plant and animal breeding populations (de et al., 2013; Huang et al., 2017a), was used to estimate the phenotypic performance in the present study.

Marker density is another important factor influencing the power of GWAS. On cotton chromosomes, the distribution of recombination rate and genes/markers showed a close association (Shen et al., 2017). Only with enough marker density, the true LD distribution and decay distance, which influence the resolution and capacity of the QTLs in GWAS, could be detected. With the release of the whole genome sequence of *G. hirsutum* (Li et al., 2015; Zhang et al., 2015), a high-density SNP chip, CottonSNP80K, was developed and verified to be a reliable, efficient, and high-throughput tool for genotyping *G. hirsutum* accessions and genome analysis (Cai et al., 2017). This SNP chip was used for genotyping of the association panel in this study and resulted in an average polymorphic marker density of 1SNP/46.267Kb genome-widely, varying from 1SNP/81.972Kb (Chr02) to 1SNP/26.252Kb (Chr16), which fulfilled the requirement for GWA mapping.

The use of appropriate statistical algorithms is also essential for GWAS. To reduce the errors related to population structure and kinship, the optimal model, MLM(Q+K) was selected by comparing six models using the quantile–quantile analysis. The results indicated that this strategy was effective. The 196 accessions were assigned to two subpopulations based on the peak of Δ*k*. The sub2 contained accessions mostly from YRR, whereas the sub1 possessed genotypes with wide geographic origins (Figure 3A). These results indicated that extensive exotic introductions or use in crosses of parents originating from diverse geographic regions in China and other cotton-growing countries contributed to gene exchange among cotton accessions (Zhao et al., 2015; Nie et al., 2016; Huang et al., 2017a). This is in general agreement with the common practice of cultivating breeding populations obtained by crossing parents with different genetic relationships and backgrounds to achieve on-going improvements in targeted traits (Hao et al., 2017). Finally, 47 significant SNPs located in 28 QTLs were identified for seven seed nutrient traits, of which 40, 44, 39, 36, and 30 loci were shared with GLM, GLM(Q), GLM(PCA), MLM(K), and MLM(PCA+K), respectively.

### LD decay in upland cotton

The previous studies showed that the LD decay influenced the resolution and capacity of GWAS, and varied among different species and populations. In *Zea mays, Glycine max, Oryza sativa*, and *Brassica napus*, the LD decay distances were <100 kb, <600 kb, <1 Mb, and <6 Mb, respectively (Hyten et al., 2007; Huang et al., 2017a). The LD decay distance also varied in upland cotton populations from 3.4 to 25 cM (Abdurakhmonov et al., 2008; Fang et al., 2013; Saeed et al., 2014). Different subpopulations showed variable LD decay speed (Cai et al., 2017; Li et al., 2017). A previous study showed that subpopulations with a rapid LD decay experience higher selective pressure during evolution (Cai et al., 2017). With the approximate ratio of 1.75 cM/Mb (Wang et al., 2015), the LD decay distance in this study would be 5.60~5.78 cM (3.20~3.30 Mb) when *r*^2^ = 0.1 in the whole genome. This result showed a slower LD decay in upland cotton, which agrees with the findings of Huang et al. (2017a). This slower LD decay may be caused mainly by the short cotton breeding history in China and the low rate of outcrossing, and possibly by the loss of genetic variation due to inbreeding and founder effect (Mackay and Powell, 2007; Li et al., 2013b; Huang et al., 2017a).

Furthermore, the estimates of LD provide insights into the haplotype block structure of the various chromosomes, providing researchers with a way to efficiently select markers and infer genotypes based on nearby loci (Reddy et al., 2017). In the study of Reddy et al. (2017), adjacent and pairwise measurements of LD were calculated and the average LD decay in *G. hirsutum* was 117 Kb, which was shorter than that calculated using multi-locus LD with 200 step windows in our study. The shorter LD decays were useful for identifying the QTL intervals that resulted in identifying less spurious candidate genes in GWAS experiments. However, we used the longer LD decay in this study in order to cover more candidate genes possibly related to the traits, which would be identified with the TM-1 gene expression database and candidate gene function or annotation analysis in future studies.

In addition, LD decays varied among chromosomes from 0.2 to 5.75 Mb in this study (Table 4; Figure S2). LD decays in Chr02, Chr03, Chr04, Chr11, Chr12, Chr19, Chr20, Chr21, Chr22, and Chr26 were relatively lower (<1.00 Mb), whereas those in Chr06, Chr08, Chr17, Chr23, and Chr25 were higher (>3.00 Mb). The chromosomes with slower LD decay might be involved in the domestication process (Li et al., 2013b), whereas the chromosomes with higher LD decay underwent frequent selection and intensive utilization in breeding (Huang et al., 2017a). In the present study, 22 of all 28 QTLs were on chromosomes with relative lower LD decay (< 2.00 Mb), which agreed with the fact that less attention had been paid to cottonseed nutrient traits compared to yield and fiber-related traits in cotton breeding practices. Homologous chromosomes Chr04 (A04) - Chr22 (D04) and Chr11 (A11) - Chr21 (D11) had similar lower levels of LD, implying that these experienced parallel evolution during the domestication process. Besides, the LD distances among the rest of the homologous chromosomes were different, indicating that a pair of homologous chromosomes had evolved differently.

### Stable and new QTLs conferring cottonseed oil and protein content

Stability of QTLs/markers across populations, environments, and genetic backgrounds is essential for MAS in breeding practices. GWAS is an efficient method to identify QTLs and dissect the genetic control of complex quantitative traits (Saeed et al., 2014; Islam et al., 2016; Cai et al., 2017; Huang et al., 2017a; Du et al., 2018). Compared to the agronomic and quality traits of cotton, very few reports in linkage mapping (Song and Zhang, 2007; Yu et al., 2012; Liu et al., 2015a), even less in GWAS (Badigannavar and Myers, 2015; Liu et al., 2015b) have been previously reported. In addition, fewer stable QTLs have been verified in earlier investigations (Yu et al., 2012; Liu et al., 2015a,b). Through comparing the physical position of the associated/linked markers aligned to TM-1 physical map using the automated batch BLASTN search with *E* ≤ 1e^−10^, the QTLs were detected herein and in previous reports (Song and Zhang, 2007; Yu et al., 2012; Badigannavar and Myers, 2015; Liu et al., 2015a,b). Of the 28 QTLs detected in this study, four QTLs (Table S8) were also detected in the previous studies (Yu et al., 2012; Liu et al., 2015a). Briefly, in the co-confidence interval of *qGhLA-c25* and *qGhPA-c25* detected herein, a seed crude oil QTL, *qOil-c25-1* (linked to SSR BNL3103), was detected (Yu et al., 2012). The *qGhTA-c12* for TA in this work shared a confidence interval with a previously mapped QTL *qOil2-c12-1* (BNL4059-BNL2717) for cottonseed oil (Yu et al., 2012). The *qGhSA-c3* for SA of the present work located about 2 Kb away from the SSR NAU3016 associated with SA (Liu et al., 2015a).

In addition to the four stable QTLs mentioned above, some new QTLs including 18 for seed oil and fatty acid composition and six for seed protein were also identified in this work and their stability needs to be verified. Besides comparison of QTLs identified in different works, candidate gene function or annotation analysis is an alternative method widely used in preliminary verification of the detected QTLs. The annotation information of the candidate genes in the QTL regions (Table S7) will no doubt contribute to the further verification of these QTLs/genes. Particularly, the candidate region of *qGhPR-c26* for PR (total protein) contained 8 genes (Gh_D12G1160 - Gh_D12G1167) annotated with *Arabidopsis thaliana*, SwissProt, InterProtscan, and GO function (Table S4). The candidate gene Gh_D12G1162 (GIF1: GRF1-interacting factor 3), which plays an important role in the governing of cell proliferation by means of cell cycle regulation and in other developmental characteristics associated with the function of shoot apical meristem, was identified in *A. thaliana* (Lee et al., 2014; Table S4). Gh_D12G1163 (KCS1), which is considered as the rate-limiting key enzyme by which the substrate and tissue specificities of fatty acid elongation are also decided in higher plants (Xiao et al., 2016), may affect the ratio of oil and protein in cottonseed. The genes, Gh_D12G1160 and Gh_D12G1161, coding the basic helix-loop-helix (bHLH) DNA-binding family proteins, which have been identified and characterized functionally in many plants with a critical role in the control of various biological processes including growth, development, and responses to various stresses (Gangappa and Chattopadhyay, 2013; Yastreb et al., 2016), may be related to the development of ovule and even to the protein accumulation during the development of seed. The genes, Gh_D12G1164, Gh_D12G1166, and Gh_D12G1167 (D-arabinono-1,4-lactone oxidase family protein), are related to oxidoreductase activity, D-arabinono-1,4-lactone oxidase activity, FAD binding, and catalytic activity in sperm cells and hypocotyls. Gh_D12G1165 encodes a plant invertase/pectin methylesterase inhibitor superfamily protein. In addition, the TM-1 gene expression database showed that three genes, Gh_D12G1162, Gh_D12G1165, and Gh_D12G1161, were expressed preferentially in the ovules (Table S4; Zhang et al., 2015). Therefore, these three genes were very likely to be involved in the protein synthesis and accumulation in cottonseeds.

In future verification studies, these candidate genes might be identified by detecting the expression of these genes during the different development stages of target regions and/or by inhibiting or overexpressing these genes.

## Author contributions

X-LS and X-ZS designed the experiments. YY and X-LS wrote the manuscript. XW, HX, LW, QW, JT, WF, and GZ helped in collecting phenotypic data. MS helped analyze the results and revised the manuscript. YY, XW, and LW performed most of the experiments and contributed equally to this work. All authors read and approved the final manuscript.

### Conflict of interest statement

The authors declare that the research was conducted in the absence of any commercial or financial relationships that could be construed as a potential conflict of interest.
