# Supplementary material for: Genome-Wide Association Study Identifies Candidate Genes Related to Seed Oil Composition and Protein Content in Gossypium hirsutum L
Source: Front Plant Sci. 2018 Oct 22;9:1359. doi: 10.3389/fpls.2018.01359 (PMC6204537; doi:10.3389/fpls.2018.01359)
Supplement: Supplementary file 2 [file Image_1.PDF]

Supplementary Figures

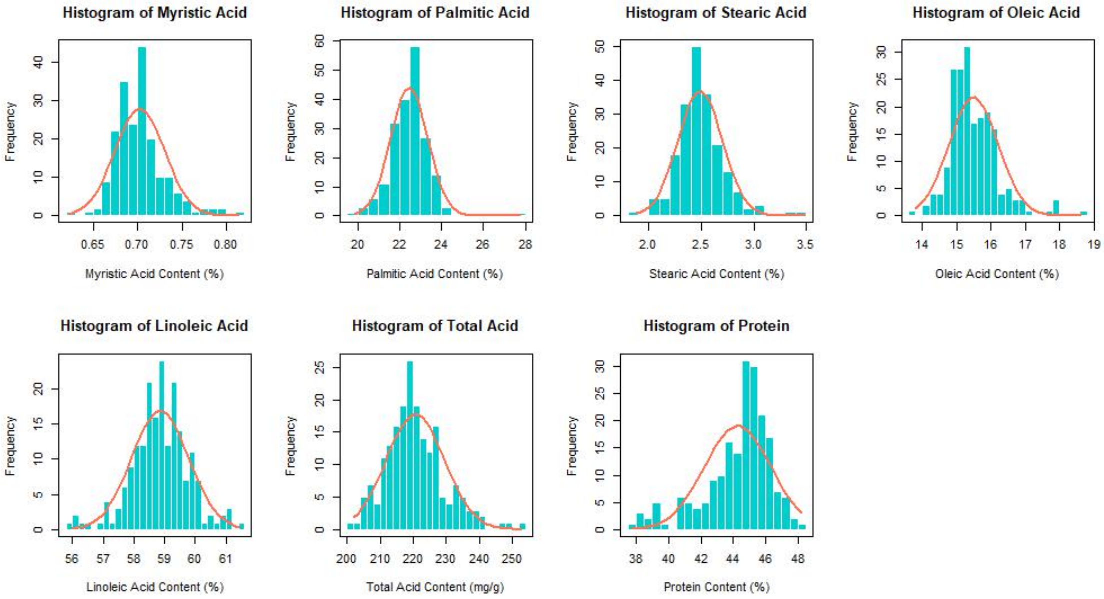

FIGURE S1 | Histogram for MA, PA, SA, OA, LA, TA and PR.

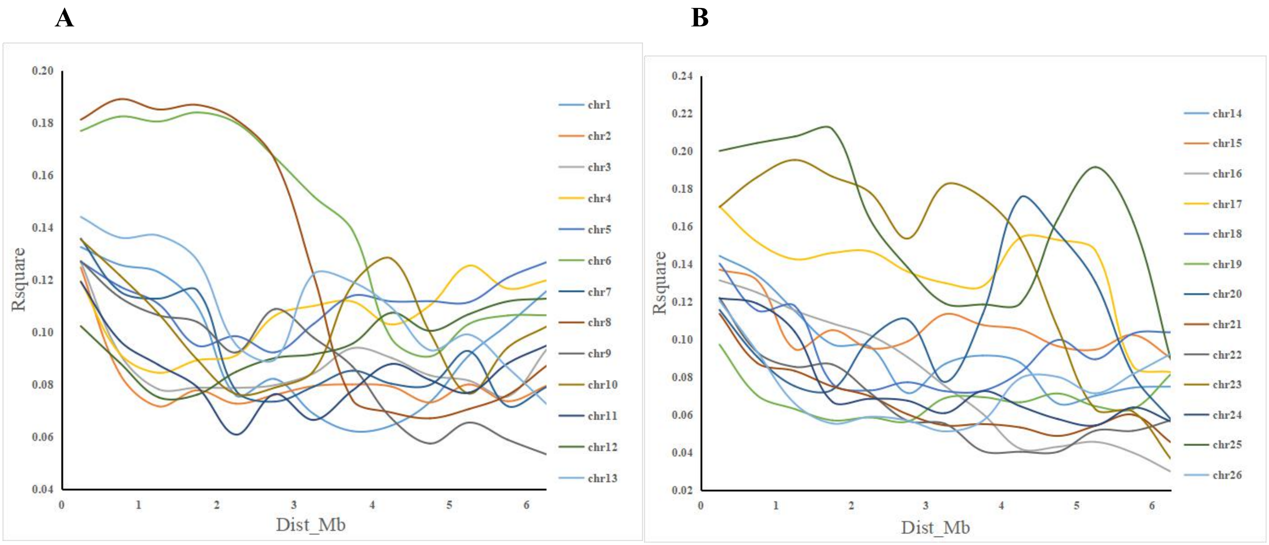

FIGURE S2 | Curve chart of LD decay for each of the 26 chromosomes. (**A**) LD decay for 13 A-genome chromosomes: Chr01 -Chr13. (**B**) LD decay for 13 D-genome chromosomes: Chr14 -Chr26.

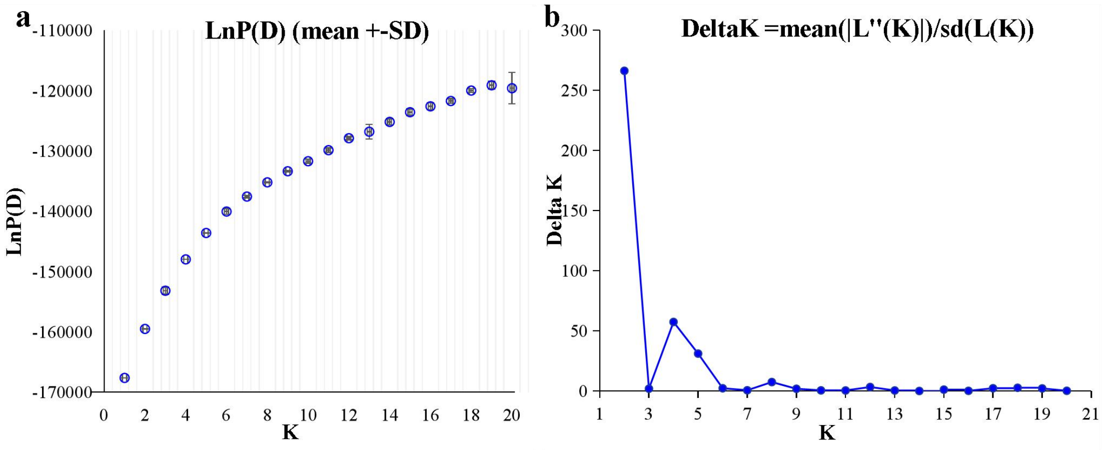

FIGURE S3 | LnP(D) and Ln(DK) values plotted from 1 to 20. (a) LnP(D) (mean +- SD) values plotted from 1 to 20. (b) Ln(DK) values plotted from 1 to 20.

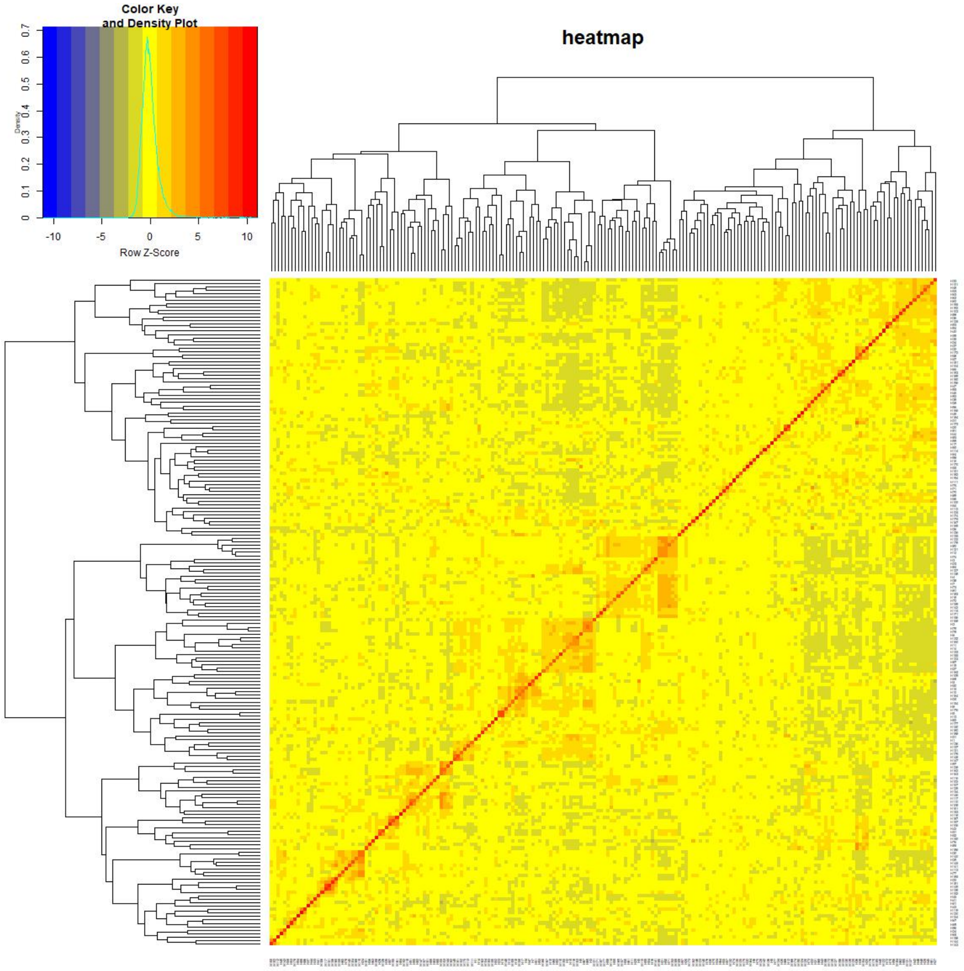

FIGURE S4 | Heatmap for Kinship analysis of 196 accessions based on genotype.
